# Pelvic Desmoid Tumor: A Rare Case with Difficult Diagnosis and Treatment

**DOI:** 10.1155/2022/7653246

**Published:** 2022-06-28

**Authors:** Mariana Ormonde, Despoina Argyropoulou, Cândida Lourenço, João Bastos, Ana Quintas

**Affiliations:** ^1^Obstetric & Gynecology Department from Hospital do Divino Espírito Santo de Ponta Delgada, Portugal; ^2^Pathology Department from Hospital Garcia de Orta, Portugal; ^3^Radiology Department from Hospital Garcia de Orta, Portugal; ^4^Urology Department from Hospital Garcia de Orta, Portugal; ^5^Gynecology Department from Hospital Garcia de Orta, Portugal

## Abstract

Desmoid tumors are rare benign neoplasms, with locally aggressive characteristics. Ongoing or previous pregnancy, antecedent trauma, and familial adenomatous polyposis are known risk factors. Still, the majority of cases are sporadic and its etiology is still unknown. These tumors may occur in any body site, but retroperitoneal and pelvic desmoid tumors are extremely rare. Nonspecific clinical and radiological findings lead to erroneous diagnosis in 50% of patients before surgery. We present a case of a young multiparous female with a deep infiltrative lesion adherent to the right pelvic sidewall leading to severe right hydroureteronephrosis and ipsilateral loss of renal function. Although deep endometriosis was suspected, malignancy features could not be excluded by imaging studies. The patient underwent an exploratory laparotomy for definite diagnosis and treatment, which led to right nephrectomy, hysterectomy, and right oophorectomy because of deep infiltration and difficult dissection. Definite histologic diagnosis revealed the presence of a pelvic desmoid tumor. Positive margins were encountered but, until this moment, no disease relapse occurred.

## 1. Introduction

Desmoid tumors are a class of benign neoplasms that, although locally aggressive, have no known potential for metastasis [1, 2] [3, 4]. These tumors are extremely rare, representing only 0.03% of all neoplasia and less than 3% of all soft tissue tumors [1, 3, 5]. They are more common in young multiparous women and the majority of cases are sporadic, with no known etiology [6–8]. Still, 5-15% are linked with familial adenomatous polyposis (FAP) [1]. Other known risk factors for this disease are pregnancy and previous trauma [3, 8]. Desmoid tumors may develop in any body site, but the majority (60%) are localized in extra-abdominal areas, especially the abdominal wall [3, 6, 8]. Retroperitoneal pelvic desmoid tumors are even rarer [7, 9, 10]. They are usually diagnosed as a deep infiltrative mass, sometimes palpable, but asymptomatic otherwise, until they cause visceral compression, leading to vascular, urinary, enteric, and/or neural manifestations [1–3, 11]. Because of the rarity of these tumors, only 50% of them are properly diagnosed before surgery. Frequent differential diagnosis encompasses ovarian neoplasms, sarcoma, GIST, endometriosis, and organized hematoma [5–7, 10]. Evidence-based treatment guidelines are nonexistent, and most data is encountered in case reports and small cohorts [5]. Expectant treatment for asymptomatic patients and stable lesion size seems reasonable [12–14]. On the other hand, surgery may be indicated for symptomatic patients, rapidly growing tumors or loss of organ function [5, 12]. Yet, even after complete resection, these tumors present a high recurrence rate (20-68%) [2–4, 7] [9].

## 2. Case Report

A 27-year-old African woman was referred to our Gynecology Department through her primary care physician, due to complaints of chronic pelvic pain and right lumbar pain. She did not have any other symptoms, such as genitourinary complaints. She had a history of loop electrosurgical excision procedure for cervical high-grade squamous intraepithelial lesion, with subsequent negative HPV testing and cytology. She denied any other previous surgeries, trauma, or other medical problems. However, during investigation, we found a not previously diagnosed arterial hypertension. She has had two previous normal deliveries (six and two years ago), and she breastfed until the previous six months. Following menarche at 12 years old, she always had regular menstrual cycles with mild dysmenorrhea. She was taking a progestogen-only pill for birth control. Family history was irrelevant concerning hereditary and oncological diseases. During gynecological examination, we did not detect any significant abnormalities. However, during rectal examination, a firm and painful nodule was felt, measuring around 2 centimeters, just below the insertion of the right sacrospinous ligament. We could not palpate this nodule nor any other anomalies during abdominal examination.

Previous to referral, an abdominal ultrasound was done, which revealed severe right hydroureteronephrosis. There were no signs of renal nor ureteral lithiasis, but diminished ipsilateral renal parenchyma was noted. Following this result, she had undergone a pelvic computed tomography (CT) which, besides the same findings, also revealed an oval, solid, and heterogeneous lesion, with 11 millimeters (mm). This lesion was fixed on the right pelvic sidewall, near the right iliac vessels, and was causing the already known compression of the right ureter. Also, this lesion seemed to be in close relation with the right ovary, which was fixed as well to the right pelvic sidewall. No other pelvic nor genitourinary anomalies were found. The radiologist suggested that these findings could be related to deep pelvic endometriosis.

**Figure 1:**
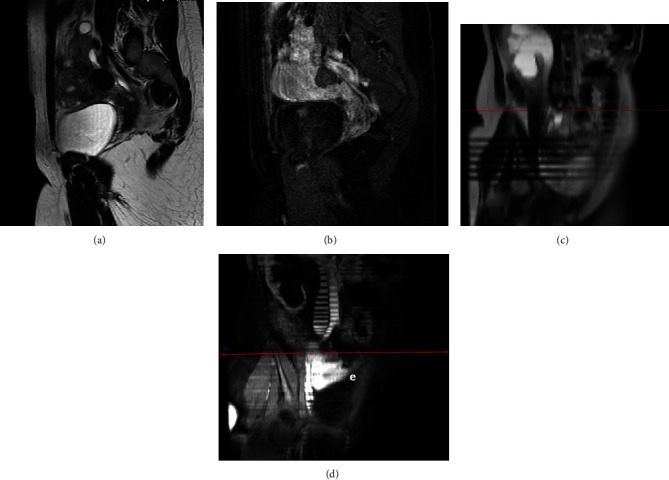
Pelvic magnetic resonance imaging showing the following: (a) sagittal T2-weighted spin-echo image: a round mass with very low homogeneous signal intensity in its central part is present; (b) sagittal 90 second postgadolinium T1-weighted fat-suppressed spin-echo image: the upper aspect of the mass enhances reflecting active disease and the nodular desmoid tumor exhibits minimal enhancement confirming its fibrous nature; (c) paracoronal T2-weighted; and (d) postgadolinium T1-weighted images showing right kidney hydronephrosis due to an extraureteral obstruction confirmed to be a desmoid tumor.

Pelvic transvaginal ultrasound was performed after the first gynecological appointment, which revealed a spiculated, infiltrative nodule with a diameter of 17 mm, lateral and adjacent to the right ovary. Still, no specific conclusion was made. Consequently, a pelvic magnetic resonance imaging (MRI) was requested (Figure 1). This exam also revealed a spiculated nodule with decreased signal on T2w images and decreased signal in T1w images. This nodule did not enhance significantly after gadolinium-based contrast and measured 28 × 22 × 29 mm. It was adjacent to the anterior part of the psoas muscle and caused right ureteral immobilization and stenosis, with consequent severe hydroureteronephrosis. This exam suggested that this lesion could be an endometriotic deep nodule. Still, invasive and aggressive characteristics could not be excluded. Taking these findings into account, histologic characterization was recommended.

Simultaneously, the patient was referred to the Urology Department, and, after the first appointment, a nuclear medicine renogram was performed. It showed severe asymmetry of the renal function, with only 19% of global renal function being provided by the right kidney. Normal left kidney function was present.

**Figure 2:**
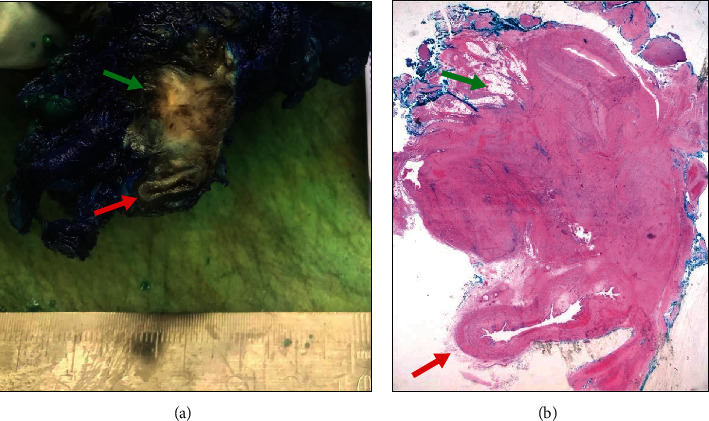
(a) Macroscopic and (b) microscopic correlation of a poorly circumscribed lesion, with a white and whorled appearance, on the cut surface, that infiltrates the surrounding soft tissue (indicated by the green arrows). The tumor compresses the adjacent ureter (indicated by the red arrow).

Considering these findings, and after a multidisciplinary decision together with urology, we decided to perform an exploratory laparotomy to establish diagnosis and treatment, which was accepted by the patient. However, we could only operate this patient nine months after the first appointment, because of hospital restrictions and lack of operating times during COVID-19 pandemic. During surgery, we identified the right ureter trapped by the known infiltrative nodule. Macroscopically, this lesion was bigger than expected, with an estimated diameter of 60 mm. The lesion was solid, firm, and infiltrative, surrounding the ureter (Figure 2), right iliac vessels, and hypogastric nerve. Also, it infiltrated the right parametrium. We did an incisional biopsy for an intraoperative diagnosis. On frozen section, a descriptive diagnosis of a nonspecific, bland fusiform cell lesion, with no cytological atypia and associated dense collagenated stroma, was established. An epithelial malignancy or endometriosis was excluded. Also, during surgery, dissection and total excision of the mass was laborious. Due to the fact that the right kidney was already impaired, the urologist decided to perform right nephrectomy. Also, because of deep infiltration, there was a need for total hysterectomy and right oophorectomy. Bilateral salpingectomy was also performed. A drain was left on the right renal fossa until day 3 after surgery. Estimated blood loss was 400 milliliters.

Postsurgical hospitalization was complicated by urinary retention and fever on day 5 after surgery. We started empiric antibiotic therapy with intravenous cefuroxime. Three days later, urine culture revealed the presence of multiresistant Proteus mirabilis and Klebsiella pneumoniae, so we changed antibiotic therapy to gentamicin. The patient had a noticeable recovery, with no other complications. She was discharged 13 days after surgery.

**Figure 3:**
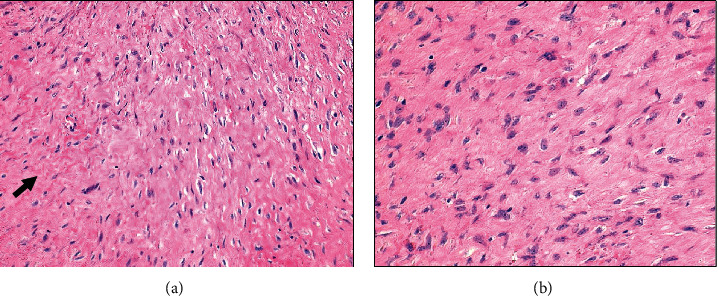
(a and b) Microscopically, the lesion consisted of a proliferation of relatively uniform spindle cells, arranged in long fascicles, with fusiform nuclei and inconspicuous nucleoli, in an abundant collagenous stroma, pointed by the black arrows. The tumor showed an infiltrative pattern of growth.

**Figure 4:**
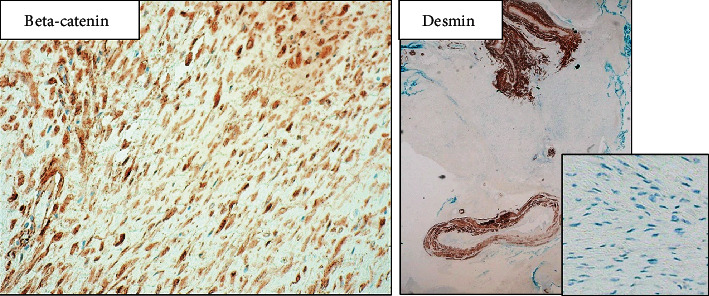
The neoplastic cells expressed positive aberrant nuclear staining for beta-catenin and were negative for desmin.

Around six weeks later, in the first postsurgical hospital visit, the patient was stable, with no pain. However, she had loss of sensitivity in the medial part of the right thigh. Vaginal vault was scarred and blood and urine analysis were normal. In the Pathology Department, on gross examination, the lesion consisted of an infiltrative, fibroelastic tumor that measured approximately 5 cm on the long axis, with a white, whorled appearance and ill-defined borders, on the cut surface (Figure 2). The lesion, macroscopically, seemed to compress the adjacent ureter. Microscopically, the tumor was composed by an infiltrative growth pattern proliferation of bland spindle cells, arranged in long fascicles, with fusiform, uniform, nuclei, and inconspicuous nucleoli, with no cytological atypia, in a stroma with bundles of collagen (Figure 3). There were no mitoses or necrosis observed. The excision was incomplete, with focal positive surgical margins. An extensive panel of immunohistochemistry was performed. The neoplastic cells stained negative for Cytokeratins, S100, SMA, desmin, STAT6, CD34, and DOG1 and expressed positivity for ϐ-catenin (nuclear expression), favouring the diagnosis of fibromatosis (Figure 4). Due to the rarity of the diagnosis and the distinct treatment implications of such diagnosis, the case was sent for a second opinion to a national expertise pathologist of mesenchymal tumors that supported the diagnosis.

This patient was then referred to the Gynecological Oncology Group for further surveillance, and a new pelvic MRI was requested, three months after surgery. The exam did not show any signs of recurrence nor other abnormalities and patient was asymptomatic at this stage.

## 3. Discussion

We describe a rare case of a young female adult with a desmoid tumor diagnosed after surgery that was causing ureteral compression and consequent renal impairment. Desmoid tumors, also called aggressive fibromatosis, are benign tumors, although some authors may consider their behavior as intermediate, because of their local aggressiveness and high recurrence rate. There are no reports of metastatic disease [2, 3, 8]. These tumors are extremely rare, occurring in only 2-4 people per million annually [11]. Most frequently, it affects patients between 20 and 35 years old [2, 7]. Also, they are more common in women, typically with ongoing or recent pregnancies [1, 6, 8]. Our case is consistent with these demographics.

In fact, one of the known risk factors for desmoid tumors is pregnancy, and these are thought to be related to high estrogen states [8]. Another known risk factor is previous trauma, which seems to be related to an altered wound healing process, after surgeries or other body injuries [5, 8]. Still, the most acknowledged risk factor is the association with FAP, which increases by 1000-fold the risk for developing desmoid tumors, in patients with Gardner syndrome and a germline mutation of the APC gene, a component of the Wnt pathway [1, 3, 6]. So, in these patients, we should look for evidence in familiar history [3], which was not present in our patient's history. Actually, the majority of cases are sporadic and multifactorial [5]. Molecular pathways are still poorly understood and still under investigation, but somatic beta-catenin gene mutations seem to occur in 50-85% of these cases [3, 5, 8].

Regarding pathophysiology, desmoid tumors represent an infiltrative fibroproliferation disorder in the fascia and muscle aponeurosis [1, 3, 10]. They may develop in any body site but most frequently appear in the anterior abdominal wall and shoulder girdle [1, 3, 8]. Retroperitoneal and specifically pelvic desmoid tumors, such as in our case, are unusual, occurring in less than 20% of cases [15].

The major problem with these lesions is their capability to expand and adhere to surrounding structures, leading to the destruction of close organs, and consequent loss of function and/or visceral and neurological symptoms [10, 12]. Even so, we can find cases in the literature with a large spectrum of disease progression, ranging from spontaneously regressing lesions to rapidly growing tumors [10, 12]. Most are asymptomatic until organ dysfunction is present [8]. When symptomatic, pain is the main complaint reported [15]. Our patient had moderate pelvic and lumbar pain, and, unfortunately, hydroureteronephrosis was only diagnosed when severe ipsilateral renal impairment was present, which is consistent with other reports of advanced renal failure at time of desmoid tumor findings [5]. Severe hydronephrosis is, in fact, a known associated complication of intra-abdominal desmoid tumors, occurring in 28% of patients, such as the cases reported by Ishikawa, et al. and Kuwabara et al. [5, 11, 16].

Kumar et al. state that only 50% of desmoid tumors are correctly diagnosed before surgery. Earlier diagnosis is difficult because of its rarity and nonspecific clinical and imaging features [6]. There must be a high clinical suspicion, especially in patients with no familiar history nor other medical problems [3, 7]. Most common differential diagnoses for intra-abdominal and pelvic fibromatosis are ovarian neoplasm, sarcoma, metastatic tumor, GIST, sclerosing mesenteritis, endometriosis, and hematoma [1, 5, 6, 10].

Preferred imaging method for diagnosis is MRI, which is considered superior to CT in defining tumor extension and its relation to surrounding structures [2, 3, 8]. On MRI, desmoid tumors are usually hypo- or isointense to muscle on T1-weighted images and hyperintense on T2-weighted images. Also, they may show moderate to intense enhancement with the administration of gadolinium, with hypointense bands due of collagen bundles [2]. These features are not diagnostic but are typical of aggressive fibromatosis [7]. In our patient, the lesion was macroscopically larger than expected, which we think is related to the waiting period until surgery.

Definitive diagnosis is always done by histologic examination [1]. There are some cases where needle biopsy is performed, but it is usually technically difficult [11]. Also, when considering differential diagnosis of malignancy, the possibility of cell dissemination should be taken into account [5]. So, in many cases like ours, surgery is the first option for definite diagnosis and treatment [12]. Microscopically, desmoid tumors are characterized by an infiltrative pattern of growth of clonal fibroblastic and myofibroblastic cell proliferation, arranged in long fascicles, with no cytological atypia or mitotic activity, in an abundant collagenous stroma. A perivascular lymphocytic infiltrate and thick walled vessels at the periphery of the lesion can be observed [2, 8]. The neoplastic cells have an aberrant positive nuclear expression of beta-catenin, encoding a molecular activating mutation of CTNNB1. The cells also show variable positivity for vimentin, smooth muscle actin (SMA), and Calponin and are negative for cytokeratins, h-caldesmon, desmin, STAT6, CD34, and S100. The main histological differential diagnosis encountered bland spindle cell lesions such as nodular fasciitis, low-grade fibromyxoid sarcoma, and solitary fibrous tumor, all of which have specific immunohistochemical and molecular signatures [3, 8].

Treatment of this condition is still controversial and is dependent on tumor site and clinical context [8]. There is still some controversy but, increasingly, a more conservative management has been recommended [4, 11]. The National Comprehensive Cancer Network (NCCN) suggests expectant management as a primary option for stable, asymptomatic, and non-life-threatening desmoid tumors [14]. Still, surgery may be the treatment of choice for suspicion of malignancy, rapidly growing tumors, loss of organ function, or symptomatic patients [5]. We can say that this was our patients' case, where there was a loss of renal function, evolving pain and uncertainty towards the diagnosis. When surgery is performed, complete resection of the lesion with surgical negative margins is aimed [8, 12]. However, in our case, positive margins were encountered. Stankiewics and Jeyadevan described that tumor-free margins are accomplished in only 50% of patients [7]. Positive margins seem acceptable when trying to prevent significant morbidity and preserve function of surrounding structures. Its relation to local recurrence is still uncertain [8].

A multidisciplinary team should be present and surgeons should balance the need for tumor excision and functional impairment of surrounding organs and nerves [9, 12]. During our patient's surgery, right nephrectomy was performed. Even though it is a major surgery, with specific risks, the right kidney was already impaired, so we consider that no additional morbidity was added in this case. Mariani et al. reported that five out of seven cases from their pelvic desmoid series needed nephrectomy during surgery [15].

We also performed hysterectomy and right oophorectomy, which was related to deep infiltration of the lesion into the right parametrium, with no possible dissection, and difficulty in proper right ovarian artery hemostasis during dissection. Even though this was a young patient, we decided that this was the best option during surgery, for complete macroscopical removal of the tumor. Also, she already had two born children. In the first appointment after surgery, the patient complained of loss of sensitivity in the medial part of the right thigh. This was probably related to the dissection of the lesion near the genitofemoral nerve. However, the nerve was preserved, so this symptom should be temporary.

Other proposed treatment options may be considered for primary treatment or recurrence. Radiotherapy should be reserved for inoperable patients and should not be used as adjuvant to surgery. Systemic therapy could also be an option for patients in which surgical morbidity would be inappropriate [8, 11]. Targeted therapies are being studied, such as sorafenib and pazopanib [8, 13]. Also, there have been some experiences with other therapies like nonsteroidal and steroidal anti-inflammatory drugs, tamoxifen, and cytotoxic chemotherapeutic drugs [3–5, 10].

There are no evidence-based clinical protocols for surveillance after treatment [5]. NCCN proposes clinical follow-up with history and physical examination every three to six months during two to three years and then annually for a prolonged time, because of possible late relapse [3, 14]. Also, patients should undergo imaging surveillance [1, 8]. We decided to surveille our patient every three months, and also, she underwent an MRI before the first follow-up visit, with no signs of recurrence. There is evidence that local recurrence is frequent and may occur in 20-60% of patients after surgery, even with negative surgical margins [2–4, 7, 9]. The majority of recurrences happen during the first two years after treatment (89%), such as reported by Fiore et al., in a multicenter cohort of 142 patients [9]. Until the writing of this report, the patient has been surveilled for six months, with no clinical nor radiologic relapse.

## Data Availability

The data supporting our study is available with the corresponding author.
